# Two new enchytraeid species from Jeju Island, Korea (Annelida, Clitellata)

**DOI:** 10.3897/zookeys.824.25544

**Published:** 2019-02-14

**Authors:** Klára Dózsa-Farkas, Tamás Felföldi, Hajnalka Nagy, Yong Hong

**Affiliations:** 1 Department of Systematic Zoology and Ecology, ELTE Eötvös Loránd University H-1117 Budapest, Pázmány Péter sétány 1/C, Hungary; 2 Department of Microbiology, ELTE Eötvös Loránd University H-1117 Budapest, Pázmány Péter sétány 1/C, Hungary; 3 Department of Agricultural Biology, College of Agriculture & Life Science, Chonbuk National University, Jeonju 561-756, South Korea

**Keywords:** *
Achaeta multisacculata
*, Enchytraeidae, *
Fridericia floriformis
*, molecular analysis, new species

## Abstract

The enchytraeid fauna of three areas in Jeju Island (Korea) was studied, and comparative morphological and molecular taxonomic examinations (based on CO1, ITS and H3 sequences) were performed on nine samples collected in 2016. Twenty-two enchytraeid species were recorded and identified. The descriptions of two new species (*Achaetamultisacculata***sp. n.** and *Fridericiafloriformis***sp. n.**) are presented in this paper. The main diagnostic features of *A.multisacculata***sp. n.** are: three pairs of pyriform glands per segment, clitellum with two “baguette-like” packages of glands, dorsal blood vessel from VII, secondary pharyngeal glands absent, oesophageal appendages well developed, two pairs of preclitellar nephridia, the reproductive organs (except the spermathecae in V) shifted one segment forward. The main features of *F.floriformis***sp. n.** are that they are large worms, have up to 2–4 chaetae in bundles, strong body wall, thick cuticle, five pairs of preclitellar nephridia, c-type coelomo-mucocytes sometimes with some refractile vesicles, chylus cells in XII–XV, sperm funnels approximately twice as long than wide, spermathecae with long ectal duct without glands, ampullae surrounded distally by about 9–12 sessile diverticula of varying size. Molecular phylogenetic analyses supported the morphological results and confirmed the status of the two new species.

## Introduction

The investigation of the previously unknown enchytraeid fauna of Korea has been continuing since 2007. Results have been published in four previous papers that yielded a total of 19 species new to science (Dózsa-Farkas and Hong 2010; Christensen and Dózsa-Farkas 2012; Dózsa-Farkas et al. 2015; Dózsa-Farkas et al. 2018). For Jeju Island, the fauna of Hallasan National Park (Mount Hallasan) was studied and published separately (Dózsa-Farkas et al. 2018). In this paper, further faunistic results from the lowland areas of Jeju Island, outside the Hallasan National Park, are presented, including two new species. The morphological studies are supplemented with molecular taxonomic analyses targeting the mitochondrial cytochrome c oxidase subunit 1 (CO1) gene, the nuclear ribosomal ITS region and the nuclear histone 3 (H3) gene, as in earlier studies (Dózsa-Farkas et al. 2015, 2018). For this, morphologically similar species and species described previously from Korea have been selected.

## Materials and methods

### Study sites

Jeju Island (Jeju Province) encompasses 1,848 km^2^ and is the largest island in South Korea. It was formed by volcanic eruptions approximately 2 million years ago. The center of its area is occupied by Mt. Hallasan. The island has a humid subtropical climate, making it warmer than the rest of South Korea. Winters are cool and dry while summers are hot, humid, and sometimes rainy. One of our study areas, Jocheon-eup, is a wetland and currently a candidate for designation as a Ramsar Wetland City, while the two other areas have relatively stronger human impact, being both popular sites for tourists.

The three study areas and nine sites within these areas are listed below. All samples were collected in 2016 by Yong Hong, similarly as in our parallel study regarding Mt. Hallasan (Dózsa-Farkas et al. 2018).

Area I: Dongbaekdongsan, Jocheon-eup

1. Loamy soil and litter layers in *Camelliajaponica* forest (33.50925°N; 126.72014°E; 185 m asl.), 18 Aug 2016

2. Loamy soil and leaf litter in *C.japonica* forest (33.50911°N; 126.72086°E; 181 m asl.), 18 Aug 2016.

3. Clayey soil, arboreal, under *C.japonica* (33.51831°N; 126.71492°E; 150 m asl.), 18 Aug 2016.

4. Silty soil and leaf litter in *C.japonica* forest (33.51831°N; 126.71081°E; 137 m asl.), 18 Aug 2016.

Area II: Seongsan Ilchulbong Tuff Cone, Seongsan-eup, Seogwipo-si

5. Loamy soil under *Euonymusjaponicus* (33.45972°N; 126.94056°E; 129 m asl.), 29 Sept 2016.

6. Clayey soil and leaf litter under *E.japonicus* (33.46008°N; 126.93789°E; 66 m asl.), 29 Sept 2016.

7. Loamy soil and litter layers under *E.japonicus* (33.46192°N; 126.93511°E; 16 m asl.), 29 Sept 2016.

Area III: Yongnuni-orum, Gujwa-eup

8. Clayey soil at the bottom of the dormant crater, meadow (33.45859°N; 126.83192°E; 193 m asl.), 26 Oct 2016.

9. Clayey soil, meadow (33.45895°N; 126.83276°E; 207 m asl.), 26 Oct 2016.

### Methods of morphological examination

Soil samples were refrigerated until processing. Worms were extracted from the soil by the wet funnel method (O’Connor 1962). Enchytraeids were first observed and measured alive, and subsequently fixed in 70% ethanol. Some of the fixed specimens were stained with borax-carmine, and then passed through an ethanol dehydration series (from 70% to absolute), mounted temporarily in clove oil, then permanently in Euparal between two coverslips. Hence the worms were observable from both sides (Schmelz 2003). All the important morphological characters were recorded in vivo, drawn and photographed [Axio Imager.A2 microscope with DIC (differential interference contrast) illumination, AxioCam MRc 5 (Zeiss) digital camera, Axiovision software]. The whole-mounted specimens were reexamined and photographed as well. In all micrographs presented in this study, the orientation of specimens is the same: the head is either on the left side or at the top of the picture.

The holotypes and two paratypes are deposited in the National Institute of Biological Resources, Korea (**NIBRIV**). The remaining paratypes (“P”, together with slide numbers) and further studied materials are deposited at the Department of Systematic Zoology and Ecology, ELTE Eötvös Loránd University, Hungary.

### Methods of molecular analysis

Genomic DNA was extracted with the DNeasy Blood & Tissue Kit (Qiagen) according to the instructions given by the manufacturer. CO1, H3 genes and the ITS region were amplified separately by PCR using the primer pairs HCO2198 (5’-TAA ACT TCA GGG TGA CCA AAA AAT CA-3’) and LCO1490 (5’-GGT CAA CAA ATC ATA AAG ATA TTG G-3’) (Folmer et al. 1994), H3a-F (5’-ATG GCT CGT ACC AAG CAG ACV GC-3’) and H3a-R (5’-ATA TCC TTR GGC ATR ATR GTG AC-3’) (Colgan et al. 1998), ETTS1 (5’-TGC TTA AGT TCA GCG GGT-3’) and ETTS2 (5’-TAA CAA GGT TTC CGT AGG TGA A-3’) (Kane and Rollinson 1994), respectively. PCRs, sequencing and phylogenetic analyses were performed as described in detail previously by Dózsa-Farkas et al. (2015). Briefly, Sanger sequencing was carried out by the LGC Genomics GmbH (Berlin, Germany), while phylogenetic analyses including the search for the best-fit model were performed with the MEGA 7 software (Kumar et al. 2016). The obtained sequences were deposited in GenBank under the following accession codes: MH124584-MH124596 (CO1), MH124597-MH124605 (H3), and MH128727-MH128735 (ITS).

## Results

### Morphological results

In total, 22 enchytraeid species belonging to seven genera were found in the samples (Table 1), among which two are new to science: *Achaetamultisacculata* sp. n. and *Fridericiafloriformis* sp. n. With the two new species described here, the Korean fauna consists of 36 recorded terrestrial enchytraeid species to date. Additionally, one terrestrial polychaete, *Hrabeiellaperiglandulata* Pižl & Chalupský, 1984, was recorded at site 8.

### Description of the new species

#### Achaeta multisacculata sp. n.

Taxon classificationAnimaliaEnchytraeidaEnchytraeidae

http://zoobank.org/BB0641AE-3012-4D55-B23E-455AF235ED33

Figs 1A–D , 2 , 3

##### Type locality.

Clayey soil, meadow (site 9), Yongnuni-orum, Gujwa-eup, Jeju Island, South Korea.

##### Holotype.

NIBRIV0000813658, slide No. 2329, adult, stained whole mounted specimen, collected on 26 Oct 2016 by Y. Hong. **Paratypes.** In total six stained adult and one subadult specimens on slides, coll. Y. Hong. NIBRIV0000813659, slide No. 2459 and NIBRIV0000813660, slide No. 2462 from type locality. P.120.1–P.120.4, slides No. 2305, 2460, 2478, 2482 from type locality. P.120.5, subadult specimen, slide No. 2464, site 8 (clayey soil at the bottom of the dormant crater, meadow; 33.45859°N; 126.83192°E; 193 m asl.), 26 Oct 2016. **Further material examined.** Two specimens for DNA analysis and four subadults and six juvenile specimens only in vivo.

##### Diagnosis.

The new species can be recognized by the following combination of characters: (1) small, slender worms (2.5–4.2 mm long and 160–220 μm wide at clitellum in vivo), segments 25–31; (2) six pyriform glands per segment in general; (3) clitellum weakly developed, interrupted middorsally and midventrally, with two elongate, “baguette-like” packages of gland cells on each dorso-lateral side; (4) dorsal blood vessel from VII; (5) pharyngeal glands at 4/5–6/7 connected dorsally, with ventral lobes and no secondary glands; (6) two pairs of preclitellar nephridia; (7) pars tumida of midgut from XII–XVI, extending over 2–3 segments, circumferal; (8) sperm funnels small, barrel-shaped, collar well developed about as wide as funnel body; (9) male pores in XI, ventro-lateral, each pore surrounded by small inconspicuous glands; (10) spermathecae free, confined to V with an asymmetrical dilation of ampulla and the ental tube ending in an oval reservoir.

##### Description.

**Figure 1.**
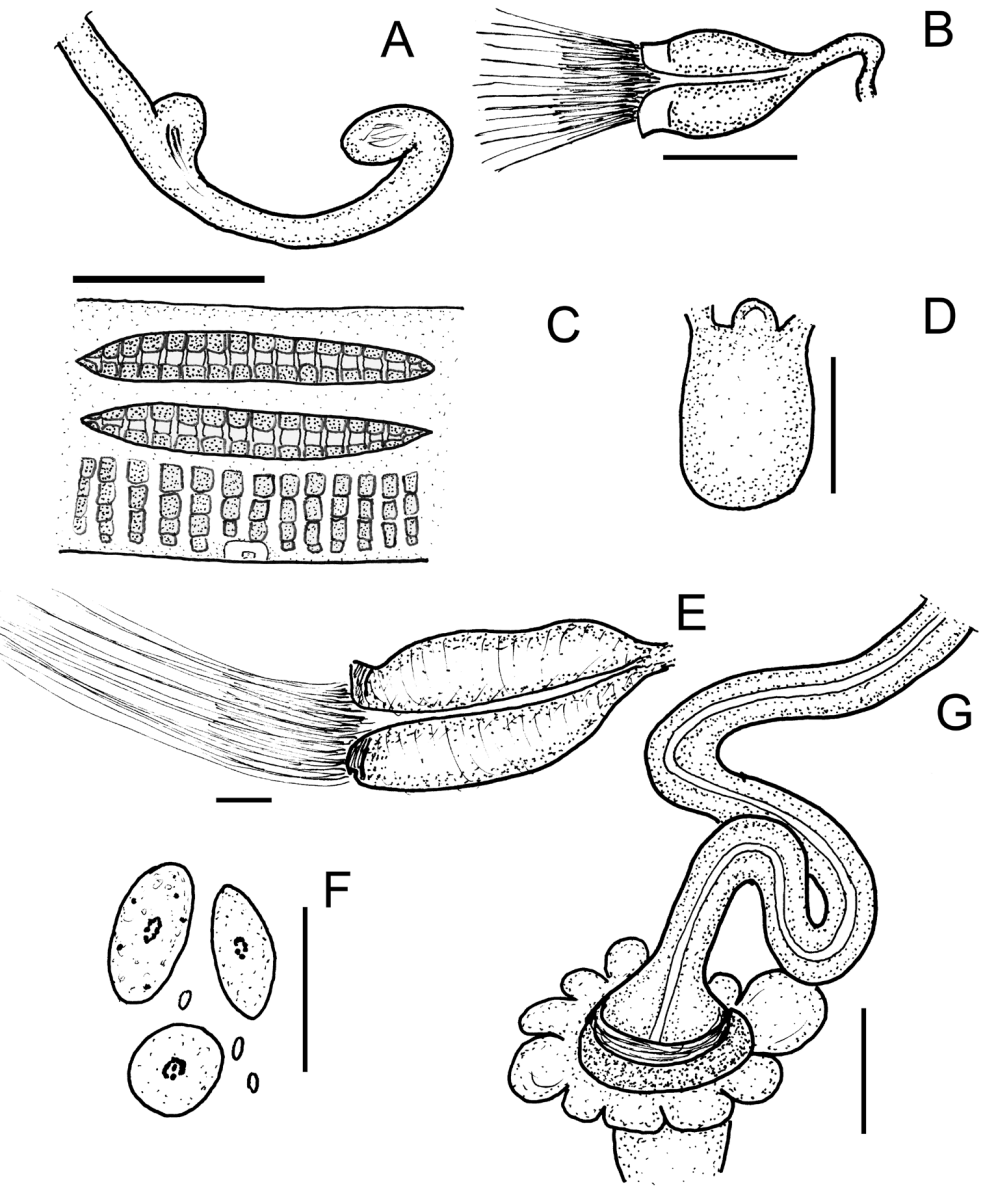
**A–D***Achaetamultisacculata* sp. n.: **A** Spermatheca **B** Sperm funnel **C** Clitellar glands, lateral view (glands middorsally and midventrally absent; two “baguette-like” packages of gland cells dorso-laterally, granular gland cells in transverse rows latero-ventrally) **D** Brain **E–G***Fridericiafloriformis* sp. n.: **E** Sperm funnel **F** Coelomocytes **G** Spermatheca.

**Figure 2.**
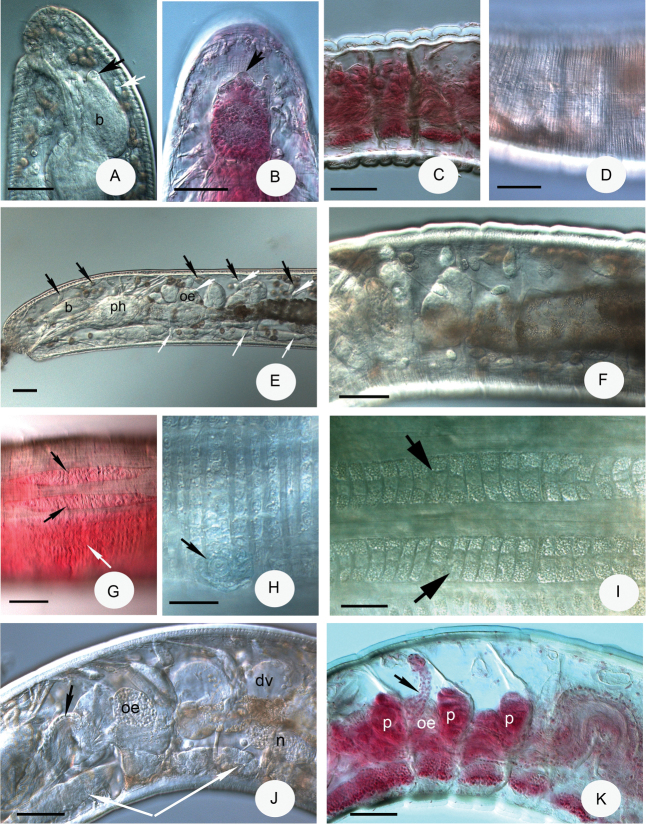
Micrograph of *Achaetamultisacculata* sp. n. **A** Head lateral view (b = brain, knob on brain marked with black arrow, first dorsal pyriform glands marked with white arrow) **B** Brain dorsal view (knob on brain marked with arrow) **C** Cuticle thicker dorsally than ventrally, lateral view **D** Transverse body wall striation by strong ring muscles **E** Forepart of body to VII, lateral view (b = brain, ph = pharynx, oe = oesophageal appendages, dorsal pyriform glands marked with black arrows, lateral pyriform glands marked with wider white arrows, ventral pyriform glands marked with narrower white arrows) **F** Pyriform glands in IV–IX lateral view **G** Clitellar glands of holotype, lateral view (dorso-laterally 2 elongate, “baguette-like” packages of hyalocytes marked with black arrows, granulocytes ventro-laterally marked with white arrow) **H** Granular clitellar glands in transverse rows ventrally, lateral view (male openings marked with arrow) **I** Two baguette-like packages of clitellar glands (marked with arrows, in the middle hyalocytes, on the margins granulocytes) **J** Segments III–VIII, lateral view (oe = oesophageal appendages with meandering canal, marked with black arrow, dv = origin of dorsal vessel, n = first nephridium, ganglia of ventral nerve cords marked with white arrows) **K** Segments IV–VIII of paratypes NIBRIV0000813659, No. 2459 lateral view (p = pharyngeal glands, oe = oesophageal glands, spermatheca marked with arrow) **A, D–F, H–J** in vivo, **B–C, G, K** fixed, stained. Scale bars: 50 μm, in **H, I**: 20 μm.

**Figure 3.**
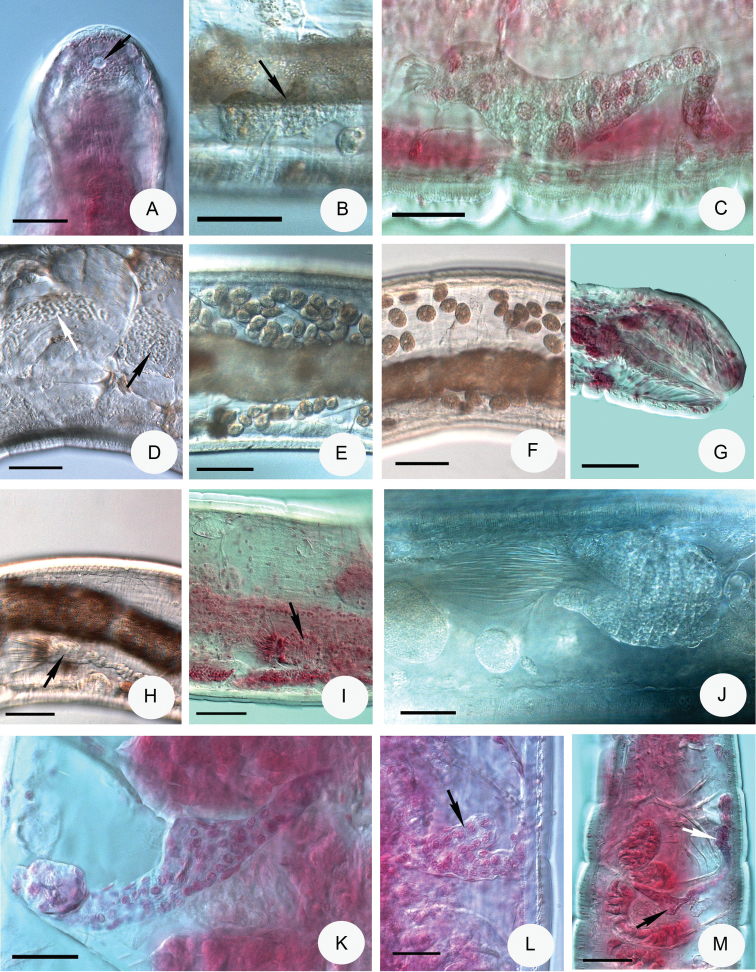
Micrograph of *Achaetamultisacculata* sp. n. **A** Head pore dorsal view (marked with arrow) **B** Preclitellar nephridia at 8/9 (marked with arrow) **C** Last nephridia at 26/27 of paratype P.120.2, No. 2460 **D** Oesophageal appendages in V (marked with black arrow), meandering canal in IV (marked with white arrow) **E–F** Coelomocytes **G** Pygidium, the anal muscles well developed **H–J** Sperm funnels **K–L** Spermathecae of paratype NIBRIV0000813659, No. 2459 (the diverticulum-like dilation of ampulla marked with arrow in L) **M** Spermatheca of paratype P 120.3 slide No. 2478 (here the ental reservoir bent back into IV marked with arrow). **B, D–E, G, I, J** in vivo **A, C, F, H, J–L** fixed, stained. Scale bars: 50 μm, in **H, I**: 20 μm.

Small, slender worm (Fig. 2E). Holotype (fixed) 3.2 mm long, 190 µm wide at VIII and 200 µm wide at clitellum (fixed), 31 segments. Paratypes 2.5–4.2 mm long, 155–200 µm wide at VIII and 160–220 µm wide at clitellum in vivo; 2.4–3.6 mm long, 150–210 µm wide at VIII and 160–210 µm wide at clitellum when fixed; segments 25–31. Head pore on prostomium (Fig. 3A). Clitellum in XI–1/2 XII weakly developed, gland cells absent dorsally and ventrally, laterally cells in transverse rows (Figs 1C, 2G, H). On each side dorso-laterally two elongate, baguette-like packages of swollen gland cells (in the middle hyalocytes, on the two margins granulocytes) narrowing at both ends (Figs 1C, 2G, I), length of baguette 190–270 μm and width 21–26 μm in the middle in vivo (100–180 μm and 20–25 μm in fixed specimens, respectively). Spermathecal pores at 4/5 in lateral position. Male pores in XI (Fig. 2H).

Body wall in vivo 10–21 μm with cuticle 5–9 μm thick dorsally and 3–5 μm thick ventrally (Fig. 2C). Ring muscles strong, resulting in transverse body wall striation (Fig. 2D). Septa 4/5–7/8 thickened (Fig. 2K). Frontal prostomial epithelium thickened ventrally. Pyriform epidermal glands (Fig. 2E, F) generally 3 pairs in dorsal, lateral and ventral position in each segment (XI also), sometimes difficult to observe or lateral, and ventral pairs absent: size variable, dorsal pairs largest, from II onwards, length 17–18 µm at II, 22–40 µm preclitellarly, 26–54 µm in the middle of body 22–26 µm posteriorly in vivo: lateral and ventral pairs from III onwards, length in vivo 11–25 µm and 11–18 µm preclitellarly, 21–24 and 15–17 µm in the middle of body, 19–25 and 12–20 µm posteriorly, respectively, but size subequal when fixed.

Brain posteriorly rounded, anteriorly convex with a conspicuous knob, 77–90 μm long, 1.6–1.8 times longer than wide in vivo (Figs 1D, 2A) (70–95 μm long and 1.5 times longer than wide, fixed, Fig. 2B). Suboesophageal ganglion of ventral nerve cord in II–IV undivided, posterior ganglia segmental and separate (Fig. 2J). Two small paired post-pharyngeal bulbs present. All pharyngeal glands at 4/5–6/7 united dorsally and with ventral lobes (Fig. 2K): first pair of glands largest, no secondary glands. Two pairs of preclitellar nephridia at 7/8–8/9 slightly constricted by septa: length ratio anteseptale : postseptale 1 : 2–3 preclitellarly, postseptale bent and tapering gradually into efferent duct, with small terminal vesicle (Fig. 3B). About 6 pairs of postclitellar nephridia (Fig. 3C) from 19/20. Dorsal blood vessel from VII (Fig. 2J), often with intensive pulsation in VII and VI, blood colourless. Coelomocytes disc-like, with fine granules, dark brown with clear nucleus, about 15–30 μm long in vivo (Fig. 3E, F) (12–16 μm, fixed). One pair of oesophageal appendages well developed dorso-laterally in V, with meandering canal in IV (Fig. 2E, J, K), clearly visible only in live worms (Fig. 3D). Chloragocytes brown, about 10–13 μm long in vivo. Midgut pars tumida inconspicuous, circumferal (i.e., not confined to ventral region of intestine), in XII–XVI (occupying 2–3 segments). Pygidium short, anal muscles developed (Fig. 3G).

Sperm funnels small, mostly barrel-shaped, 42–65 μm long in vivo (26–42 μm, fixed), about 1.5–2 times longer than wide, collar distinct 8–10 μm high, about as wide as diameter of funnel body (Figs 1B, 3H–J). Sperm ducts about 6 μm thick in vivo (4–5 μm, fixed). Spermatozoa with unusual strong tails (Fig. 3H), 50–70 μm long, heads 15–22 μm long in vivo (26–42 μm and 11–14 μm, fixed). Seminal vesicle absent. Male copulatory organs small oval, widely separated ventro-laterally, pore surrounded by small inconspicuous glands (Fig. 2H). Spermathecae free, confined to V (in one case extending into VI in vivo and in one case bent backwards into IV (slide 2478) (Fig. 3M). Ectal ducts 30–32 μm long and 14–16 μm wide in vivo (31–42 μm long and 9–10 μm wide, fixed), ducts slightly widen out to a dilation of ampullae with a diverticulum-like protrusion (dilation diameter 20–25 μm). After dilation, ental tubes (about 40–65 μm long and 20 μm wide) end in an elongated reservoir (24–45 μm long, 15–26 μm wide in vivo) (20–27 μm long and 13–18 μm wide, fixed) (Figs 1A, 3K–M).

Although the specimens are adult, the clitellar glands appear weakly developed. The reason is that this organ is fully developed only just before the release of an egg (as was remarked by Schmelz et al. 2008), and indeed our worms did not have mature eggs.

##### Etymology.

Named after the high number of ‘pyriform glands’ (*sacculus* = saccule, Latin).

##### Molecular data.

Sequences deposited in GenBank: MH128727-MH128728 (ITS), MH124584-MH124585 (CO1).

##### Distribution.

In South Korea, at sites 8–9, Jeju Island, Yongnuni-orums, clayey soil, meadows.

##### Morphologically similar species.

Two *Achaeta* species with six pyriform glands per segment have been previously described: the European *Achaetaaberrans* Nielsen & Christensen, 1961 and the South American *Achaetapiti* Bittencourt, 1974, emended Schmelz et al. 2008. The new species can be easily distinguished from *A.aberrans* which has fewer segments, 20–23 (vs. 26–31 in the new species), dorsal vessel originating in VI (vs. in VII), oesophageal appendages small and only in V (vs. well developed in IV–V), male opening in XII (vs. in XI), coelomocytes oval, finely granulated and at one end tapering into a thin process (vs. discoid and brown), the preclitellar nephridia in 6/7 and 7/8 (vs. 7/8 and 8/9), the spermathecae, when present, with laterally symmetrical ampullae. The other species, *A.piti* is very similar to *A.multisacculata* sp. n., because of the reproductive organs (except the spermathecae) shifted one segment forward, the oesophageal appendages well developed with canal in IV-V, two pairs of nephridia in 7/8–8/9, dorsal blood vessel origin in VII and the spermatheca with ectal asymmetry. In addition, in both species two elongate, dorso-lateral „baguette-like” packages of hyalocytes occur in the clitellum. Characters that differentiate *A.multisacculata* sp. n. from *A.piti* are: (1) body size slightly smaller: 2.5–4.2 mm in vivo and 2.2–3.6 mm, fixed, 160–220 μm wide at clitellum [according to Schmelz et al. 2008, live *A.piti* worms are ca. 5 mm long and 150 μm wide in vivo (fixed type specimens are 3.5–5 mm long, 190 μm wide and specimens at Zoological Museum in Hamburg even longer, 4.5–6.5 mm and up to 250 μm wide)]; (2) segment number smaller, 26–31 (vs. 31–36); (3) brain anteriorly with a conspicuous knob (vs. without knob); (4) coelomocytes dark brown, 15–30 μm long (vs. pale, 15–18 μm long, cells somewhat darker than coelom); (5) sperm funnel smaller, mostly barrel-shaped, without vesicles, 42–65 μm long in vivo, 26–42 μm, fixed, only 1.5–2 times longer than wide (vs. cylindrical, with large vesicles, ca. half as long as body diameter, more than 3 times longer than wide); (6) pars tumida of midgut at XII–XVI, 2–3 segments long (vs. XX–XXIV); (7) in pygidium anal muscles clearly visible (vs. not strongly developed); (8) spermatheca similar but except one specimen always confined in V (vs. ampulla extending into VI or VII).

#### Fridericia floriformis sp. n.

Taxon classificationAnimaliaEnchytraeidaEnchytraeidae

http://zoobank.org/1A44BB5D-9D8D-49B2-BCAD-E86D27A7B5AA

Figs 1E–G , 4 , 5

##### Type locality.

Clayey soil, meadow (site 9), Yongnuni-orums, Gujwa-eup, Jeju Island, South Korea.

##### Holotype.

NIBRIV0000813661, slide No. 2437, adult, not stained, whole mounted specimen, collected on 26 Oct 2016 by Y. Hong.

##### Paratypes.

In total 18 adult stained and not stained specimens on slides and eight specimens in 70% ethanol, coll. Y. Hong. NIBRIV0000813662, slide No. 2293, DNA 1133, adult stained, whole mounted specimen from type locality. NIBRIV0000813663, slide No. 2427, from site 8 (clayey soil at the bottom of the dormant crater, meadow; 33.45859°N; 126.83192°E; 193 m asl.), 26 Oct 2016. P.121.1-P.121.14, slides No. 2291, 2314, 2332–2333, 2389, 2428–2432, 2436, 2438, 2440, 2481 from type locality. P.121.15–121.17, slides No. 2295, 2434, 2439 from site 8 (four specimens: slide 2434, 2436, 2438 and 2481 were not stained). P.121.18, five specimens in 70 % ethanol from type locality; and P.121.19, three specimens in 70 % ethanol from site 8.

##### Further material examined.

Four juvenile and five adult specimens only in vivo (one of the whole, adult specimens was processed with molecular analysis, DNA 1136). One additional specimen in vivo and for molecular analysis (DNA 1088) from Mt. Hallasan, Jeju Island (Gwaneumsa trail, 33.41667°N, 126.55000°E, 634 m asl., 26 Oct 2016, coll. Y. Hong), referred as ‘*Fridericia* sp. 2’ in Dózsa-Farkas et al. (2018).

##### Diagnosis.

The new species can be recognized by the following combination of characters: (1) large size (body length 14–20.5 mm in vivo), segments 48–65; (2) lateral chaetae often absent, maximum 2 per bundle, ventrally maximum 3–4 chaetae per bundle; (3) clitellum well developed, between bursal slits and before the male apparati only granulocytes; (4) body wall strong and cuticle thick (3–5 μm); (5) five preclitellar pairs of nephridia; (6) coelomo-mucocytes c-type occasionally with some refractile vesicles, lenticytes scarce and small; (7) dorsal vessel from XV–XVIII; (8) chylus cells in XII–XV, occupying 2–3 segments; (9) bursal slit longitudinal slightly bent, with small transverse extensions; (10) seminal vesicle not brown; (11) subneural glands absent; (12) sperm funnel approximately as long as half body diameter, collar narrower than funnel diameter, spermatozoa 400–580 μm long, heads 100–150 μm in vivo; (13) spermatheca with 9–12 sessile diverticula of varying size mostly without sperm in them, ectal duct long without ectal glands and ampulla entally openings separately into oesophagus.

##### Description.

**Figure 4.**
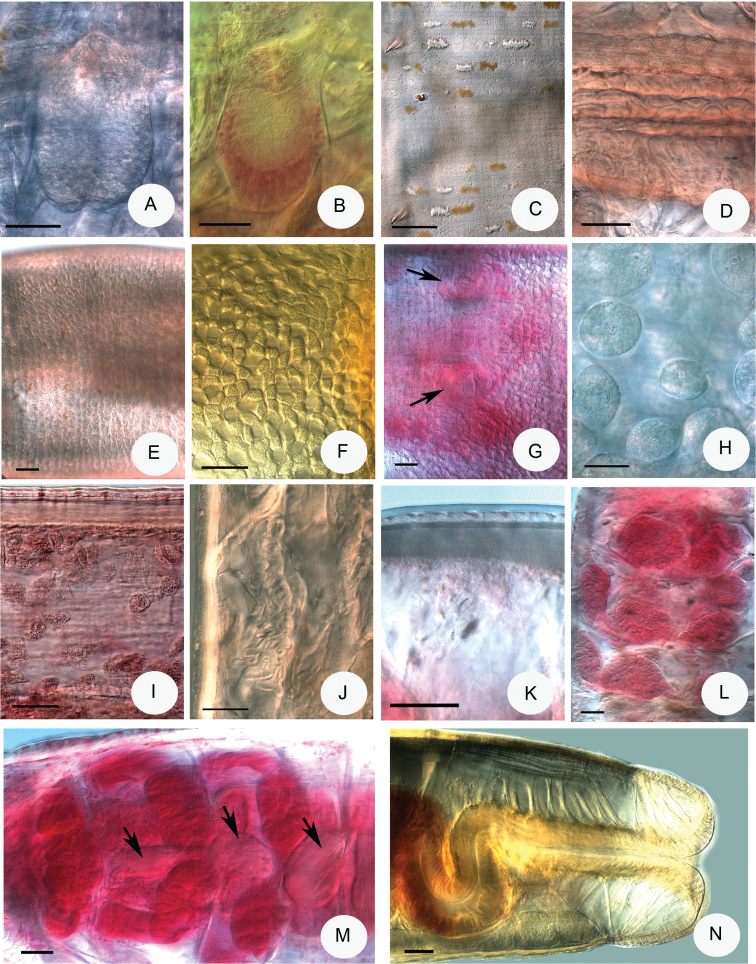
Micrograph of *Fridericiafloriformis* sp. n. **A–B** Brain (**B** paratype P. 121.4, slide 2389) **C** Epidermal glands **D** Chylus cells in XII **E–F** Clitellar glands dorsal view **G** Clitellar glands ventrally, male copulatory organs of paratype P.121.7 slide No. 2429 (marked with arrows) **H–I** Coelomocytes **J** Oesophageal appendage **K** Body wall with strong longitudinal muscles and cuticle **L–M** Pharyngeal glands (**L** paratype P. 121.15 slide No. 2295 **M** paratype P121.8 slide No. 2430 dorsal vessel marked with arrows) **N** Pygidium with well-developed anal muscle, paratype 121.12 slide No. 2438. **A, C–E, H, J** in vivo **B, G, I, K–M** fixed, stained **F, N** fixed, not stained. Scale bars: 50 μm, in **H**: 20 μm.

Large, whitish, stiff worms. Holotype 15.3 mm long, 470 μm wide at VIII and 550 μm at the clitellum (fixed), 59 segments. Body length of the paratypes 14–20.5 mm, width 400–530 μm at VIII and 500–640 μm at the clitellum in vivo. Length of fixed specimens 8–17.3 mm, width 470–580 μm at VIII and 500–620 μm at the clitellum. Segments 48–65. Chaetal formula: 1,2,(0) – 2,0,1,2 : 2,3,4 –(4),3,2,1. The inner chaetae being shorter and thinner than the outers: 30–35 × 2.5–3 μm and 54–63 × 5–6 μm (in preclitellar bundles). In the bundles with 2 chaetae the length of chaetae is different, in those with 3 chaetae one chaeta longer and the other two shorter. After the clitellum in lateral bundles of the middle part of body the chaetae mostly absent but at posterior body-end again occur 1 or 2 chaetae per bundle, length about 59–63 × 4.5–7 μm. Head pore at 0/I. Dorsal pores from VII; 2–3 transverse rows of hyaline epidermal gland cells per segment and in addition more transverse rows of dark yellow glands (visible only in vivo) (Fig. 4C). Clitellum in XII–1/2XIII, well developed, girdle-shaped, hyalocytes and granulocytes arranged in indefinite rows or reticulate pattern (Fig. 4E, F), between bursal slits and before the male apparati only granulocytes (Fig. 4G). Body wall strong, thickness about 40–54 μm, cuticle thick about 3–5 μm in vivo and fixed (Fig. 4I, K), in forepart slightly stronger than at the body end.

Brain egg-shaped, about 140–180 μm long, about 1.5–2 times longer than wide in vivo (Fig. 4A) and 120–150 μm long and 1.4–1.7 times longer than wide in the fixed specimens (Fig. 4B). Oesophageal appendages long with many branches at the end in V (Fig. 4J). All pharyngeal glands with ventral lobes, those in 4/5 united dorsally, those in the 5/6 weakly united or unconnected dorsally and those in 6/7 unconnected dorsally but occasionally weakly united (Fig. 4L, M). All septa at 5/6–9/10 thickened. At anal region the radial gut dilator muscles well developed (Fig. 4N). Chloragocytes from V, 12–26 μm long in vivo. Dorsal vessel from XV–XVIII, (in one case in XIX), blood colourless. Midgut pars tumida in XXVI–XXXII occupying 4–7 segments (only in two specimens were visible). Five pairs of preclitellar nephridia from 6/7 to 10/11, length ratio anteseptale : postseptale 1 : 2–2.5, adseptal origin of the efferent duct. Coelomo-mucocytes c-type, rounded or elliptic, sometimes with some refractile vesicles, length 28–44 μm in vivo (Figs 1F, 4H), in the fixed worms with granules and 15–28 μm long (Fig. 4I). Lenticytes scarce, small 4–7 μm long. Chylus cells in XII–XV, occupying 2–3 segments (Fig. 4D).

**Figure 5.**
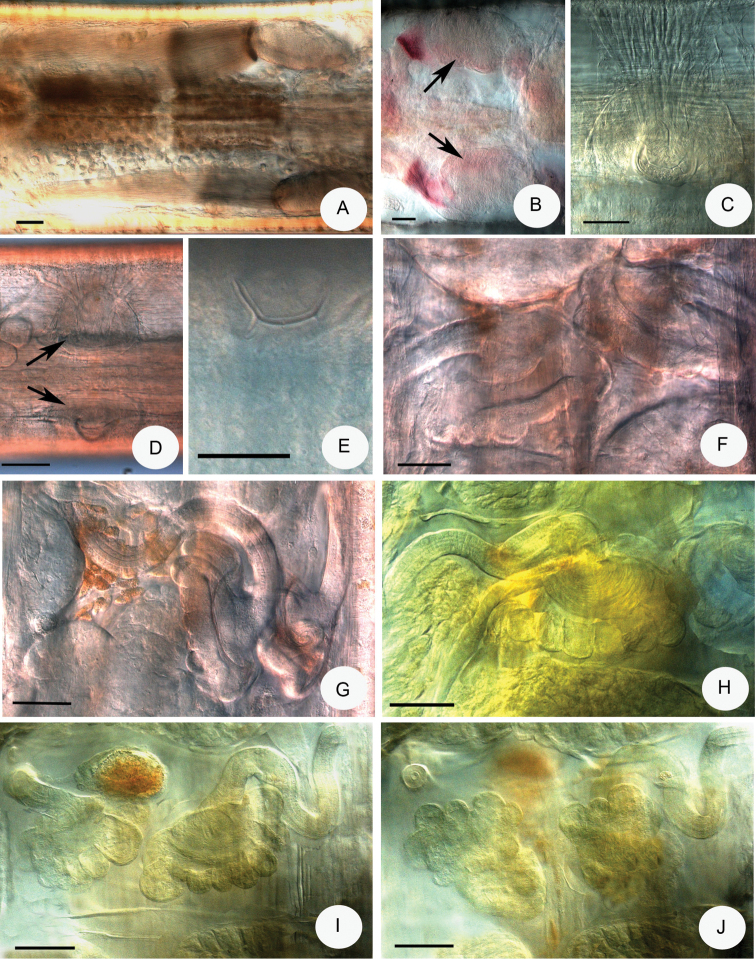
Micrograph of *Fridericiafloriformis* sp. n. **A** Sperm funnels with very visible long spermatozoa **B** Sperm funnels of paratype 121.15 slide No. 2295 (marked with arrows) **C** Male copulatory apparati, with well-developed muscle of paratype P.212.16 slide No. 2434 latero-ventral view **D** Male copulatory organs, ventral view (marked with arrows) **E** Bursal slit **F–J** Spermathecae (holotype NIBRIV0000813661, slide No. 2437, **I–J** Spermathecal ampullae of paratype P. 121.16 slide No. 2434 where the ampullar diverticula are visible on all sides around the ampullae). **A, D–G** in vivo **B** fixed, stained **C, H–J** fixed, not stained. Scale bars: 50 μm.

Seminal vesicle in XI, not brown. Sperm funnels cylindrical (Figs 1E, 5A), about 180–330 μm long and about 2 times longer than wide (in vivo). Funnel length in fixed specimens 100–220 μm, funnel body 1.2–1.8 times longer than wide (Fig. 5B); collar narrower than funnel body. The length of spermatozoa 400–580 μm, heads 100–150 μm in vivo (Fig. 5A), in fixed specimens spermatozoa 200–360 μm long and sperm heads 70–80 μm. Diameter of sperm ducts 9–10 μm in vivo, (7.5–8 μm, fixed). Male copulatory organs 130–170 μm long, 60–140 μm wide and 70–80 μm high, fixed (Figs 4G, 5C–D), retractor muscles conspicuous (Fig. 5C). Bursal slits longitudinal, slightly bent, with small additional transverse extensions (Fig. 5E). Subneural glands absent. Spermathecae (Figs 1G, 5F–J): no ectal gland, ectal ducts long, about 360–500 μm and 20–25 μm wide, canal 5–6 μm wide in vivo (250–500 μm long, 20–25 μm wide, canal 5 μm, fixed), not widened entally, projecting into ampulla, ental bulbs about 40–55 μm wide, fixed. Ampullae are surrounded distally by about 9–12 sessile diverticula of varying size : length 24–45 μm (fixed). Sperm in a circle in lumen of ampullar distal part. Diameter of ampulla and diverticula together 110–150 μm, mostly no sperm in the diverticula. Separate openings into oesophagus dorso-laterally. 1–4 mature eggs at a time.

##### Etymology.

Named after the shape of the spermathecal ampulla (more diverticula), which resembles a flower (*flos, floris*= flower, and *formis* = shaped as, Latin).

##### Molecular data.

Sequences deposited in GenBank: MH128729-MH128733 (ITS), MH124586-MH124589 (CO1), MH124597-MH124598 (H3).

##### Distribution.

In South Korea, at sites 8 and 9, Jeju Island, Yongnuni-orums, clayey soil, meadows.

##### Morphologically similar species.

**Figure 6.**
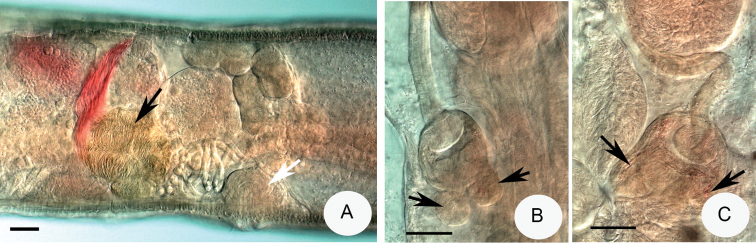
Micrograph of a *Fridericiacallosa* specimen which has spermathecae with diverticula, collected from Siberia in 1994 (Christensen and Dózsa-Farkas 1999) fixed and stained on slide. **A** X–XII (sperm funnel marked with black arrow, male copulatory apparati marked with white arrow, lateral view) **B–C** Spermathecae (diverticula marked with arrows lateral view).

There are only three species (*F.paraunisetosa* Xie et al., 2000, *F.ventrochaetosa* Nagy, Dózsa-Farkas & Felföldi, 2018 and *F.callosa* Eisen, 1878) among all *Fridericia* species, which possess more diverticula of spermathecae and the lateral chaetal bundles absent or incomplete, varying with 0, 1 or maximum 2 chaetae. *Fridericiaparaunisetosa* can easily be distinguished from *F.floriformis* sp. n. based on the following characters: smaller size (5.0–7.8 mm long vs. 8–17.3 mm, fixed), lateral chaetal bundles absent, ventrally only one chaeta per bundle (vs. 2–4 chaetae ventrally and 0–2 laterally), dorsal pores only from XVIII (vs. from VII), brain incised anteriorly (vs. convex), oesophageal appendages stout and unbranched (vs. with branches at the end) (Xie et al. 2000). *Fridericiaventrochaetosa* could be distinguished from the new species by the total absence of the lateral chaetae and having spermathecal diverticula with stalk (vs. sessile) (Nagy et al. 2018). The new species is similar to *F.callosa* in most traits (e.g., body size, segment number, strong body wall, thick cuticle, chaetal arrangement, number of preclitellar nephridia, position of chylus cells, the length of sperm), but the main differences between the two species are: in *F.callosa* the collar of sperm funnel not narrower than funnel body (Fig. 6A) (vs. narrower, Fig. 5A, B), seminal vesicle 2–3 segment large (vs. only in XI and not conspicuous). The spermathecae very variable in *F.callosa* (probably species complex) with or without diverticula, and the maximum number of diverticula is 6 (Eisen 1878, 1879; Christensen and Dózsa-Farkas 1999; Schmelz 2003). From the material collected in Siberia in 1994, some stained slides were prepared now. On these slides, it was visible that the few diverticula are oriented towards the proximal ampullar part (Fig. 6B–C) in contrast to the spermathecae of the new species, which always have many diverticula or diverticula-like protrusions surrounding the ampullae (Fig. 5F–J).

### Results of molecular analysis

**Figure 7.**
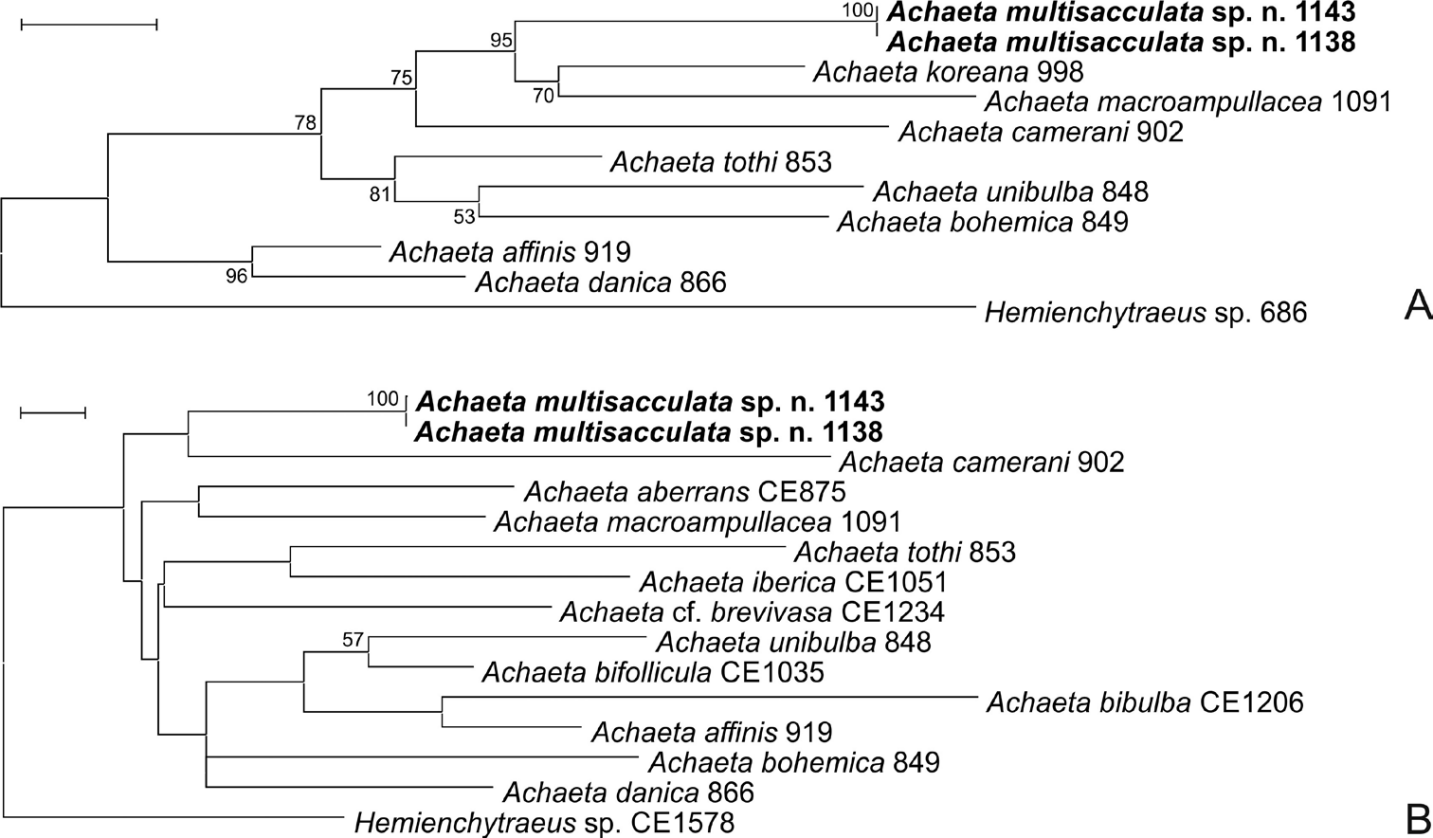
Maximum likelihood (ML) trees of studied *Achaeta* species based on the ITS region (**A**) and CO1 (**B**) gene. Bootstrap values greater than 50 are shown at the nodes. Sequences from new species described here appear in bold. **A** ML tree of the ITS region based on 736 nucleotide positions using the K2+G substitution model **B** ML tree of the CO1 gene based on 543 nucleotide positions using the GTR+G+I substitution model. Scale bars: 0.1 substitutions per nucleotide position.

**Figure 8.**
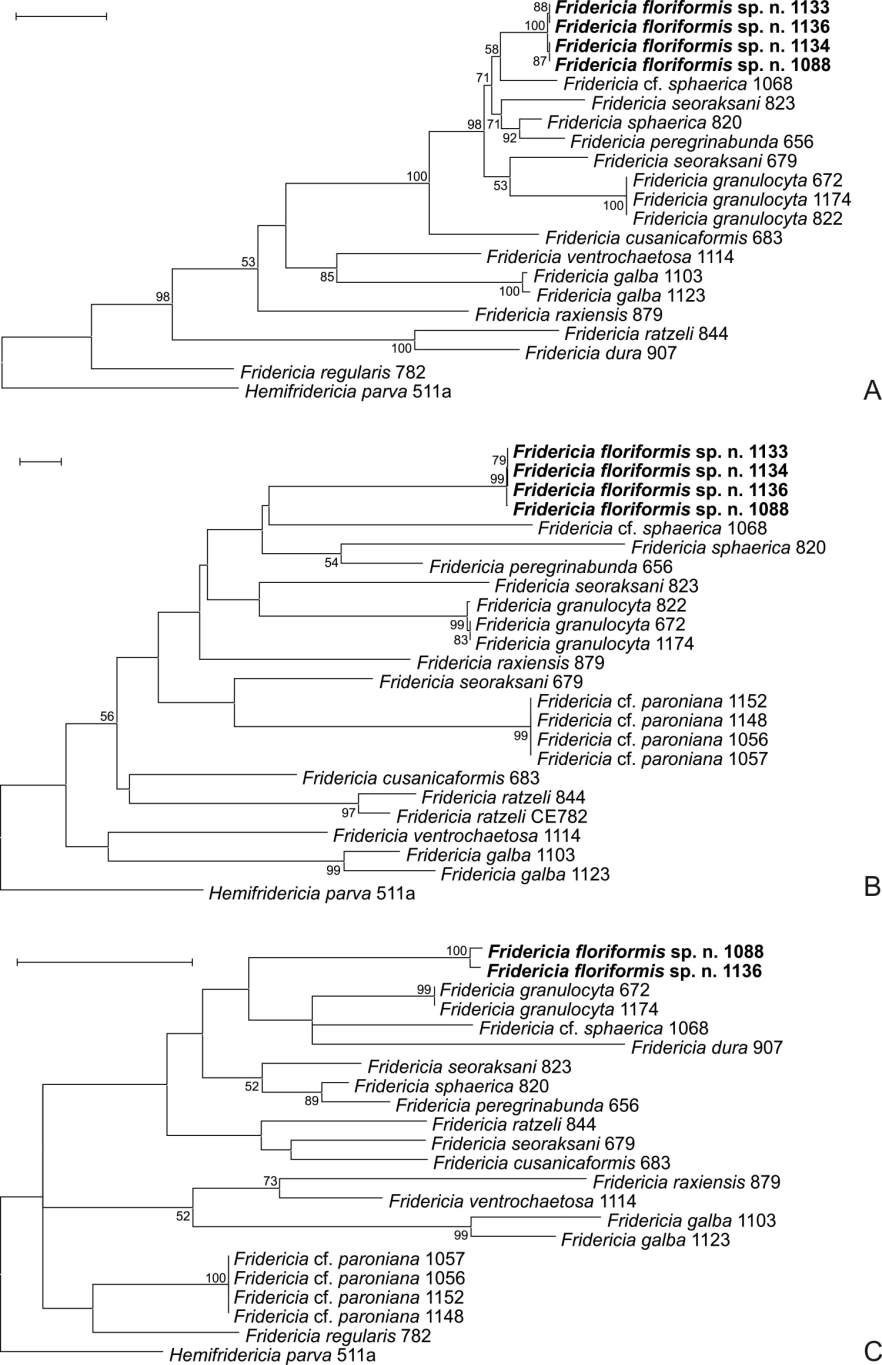
Maximum likelihood (ML) trees of studied *Fridericia* species based on the ITS region (**A**), CO1 (**B**) and H3 genes (**C**). Bootstrap values greater than 50 are shown at the nodes. Sequences from new species described here appear in bold. **A** ML tree of the ITS region based on 634 nucleotide positions using the K2+G+I substitution model **B** ML tree of the CO1 gene based on 455 nucleotide positions using the T93+G substitution model **C** ML tree of the H3 gene based on 145 nucleotide positions using the K2+G substitution model. Scale bars: 0.1 substitutions per nucleotide position, except H3 gene 0.05.

In total, 9, 13 and 9 new sequences were determined from various *Achaeta* and *Fridericia* species in the case of ITS, CO1 and H3, respectively. Additional sequences determined in previous studies (Erséus et al. 2010; Dózsa-Farkas et al. 2015, 2018; Dózsa-Farkas and Felföldi 2017, 2018; Nagy et al. 2018) were also used for comparison (Table 2). However, unfortunately, we failed to amplify the H3 gene from specimens of *Achaetamultisacculata* sp. n., which was probably due to the improper hybridization of PCR primer sequences to the extracted genomic DNA. Results of the molecular analyses confirmed that the two new species are genetically separate from morphologically similar species and species described previously from Korea and their sequences form distinct lineages on the phylogenetic trees (Figs 7, 8). This was also supported by interspecific sequence distances, since in the case of the two new species these values were similar to the interspecific sequence distances of other species involved in the analysis: 18.3-25.5% and 15.5-33.2% (*Achaeta*CO1), 42.2-66.9% and 22.3-66.6% (*Achaeta* ITS), 18.0-25.0% and 16.4-27.4% (*Fridericia*CO1), 9.1-46.3% and 6.1-56.6% (*Fridericia* ITS), 8.6-17.6% and 2.9-23.3% (*Fridericia*H3).

## Discussion

Earlier we studied and described the enchytraeid fauna of Hallasan National Park (Mt. Hallasan) from Jeju Island (Dózsa-Farkas et al. 2018). In the present study, we investigated nine new samples collected from other areas of Jeju Island. This time 22 enchytraeid species were found, two of which are new to science. According to the studied samples, sites 8 and 9 (Youngnuni-orum) were the most species-rich (with ten and seven detected species), harboring the two new species, *Achaetamultisacculata* sp. n. and *Fridericiafloriformis* sp. n. (both new species were found only in this area) (Table 1). This could be explained probably with the meadow habitat, since the other samples were collected from forest habitats. Results of molecular analyses confirmed the status of the two new species.

**Table 1.** Detected enchytraeid species (and the polychaetea) and their distribution at the study sites. New species described here are highlighted in bold, while new species from Jeju Island described earlier in a previous paper (Dózsa-Farkas et al. 2018) are marked with an asterisk, species new to science from Korea with #.

	Dongbaekdongsan, Jocheon-eup 18.08.2016				Seongsan Ilchulbong Tuff Cone 29.09.2016			Youngnuni-orum, Jeju 26.10.2016	
Species site code	1	2	3	4	5	6	7	8	9
*Achaetamacroampullacea* Dózsa-Farkas et al., 2018 *^, #^								+	+
***Achaetamultisacculata* sp. n.**								+	+
*Enchytraeusbuchholzi* Vejdovský, 1878 *sensu lato*	+				+	+	+	+	+
*Enchytraeuschristenseni* Dózsa-Farkas, 1992						+	+		
*E.dichaetus* Schmelz & Collado, 2010								+	+
*Fridericiacusanicaformis* Dózsa-Farkas et al., 2015 *^, #^									+
*Fridericiacf.sphaerica* Dózsa-Farkas et al., 2015 *^, #^						+			
*Fridericiaseoraksani* Christensen & Dózsa-Farkas, 2012 ^#^								+	
*Fridericiabulboides* Nielsen & Christensen, 1959								+	
*Fridericia* sp.					+				
*Fridericiagranulocyta* Dózsa-Farkas et al., 2015 *^, #^					+	+	+		
*Fridericiacf.paroniana* Issel, 1904								+	
***Fridericiafloriformis* sp. n.**								+	+
*Hemienchytraeusjeonjuensis* Dózsa-Farkas & Hong, 2010 ^#^	+	+			+				
*Hemienchytraeusquadratus* Dózsa-Farkas & Hong, 2010 ^#^	+	+	+	+					
*Hemienchytraeuskoreanus* Dózsa-Farkas & Hong, 2010 ^#^	+								
*Hemifridericiaparva* Nielsen & Christensen, 1959						+			
*Henleacf.ventriculosa* (Udekem, 1854)					+	+	+	+	
*Henleaperpusilla* Friend, 1911									+
*Xetadrilusjejuensis* Dózsa-Farkas et al., 2018 *^, #^		+						+	
*Xetadrilusaphanoides* Dózsa-Farkas et al., 2018 *^, #^		+							
*Xetadrilusaphanus* Schmelz et al., 2011		+							
**Enchytraeid species number (total: 22)**	4	5	1	1	5	6	4	10	7
*Hrabeiellaperiglandulata* Pižl & Chalupsky, 1984								1	

Four species (*Achaetamacroampullacea*, *Xetadrilusjejuensis*, *X. aphanoides, Fridericia*cf.paroniana) which were described from Mt. Hallasan previously, were found also in the lowland areas of Jeju Island. *Xetadrilusaphanus* did not occur in the Hallasan National Park, so the present record from Dongbaekdongsan, Jocheon-eup (site 2) is new for the Korean fauna. The comparison of the three *Xetadrilus* species (a genus established by Schmelz et al. 2011) is given in Dózsa-Farkas et al. (2018, Table 2). Six other species (*Fridericiacusanicaformis*, *F.seoraksani*, *F.granulocyta*, *Hemienchytraeusjeonjuensis*, *H.quadratus*, *H.koreanus*) described originally from other parts of Korea (Dózsa-Farkas and Hong 2010; Christensen and Dózsa-Farkas 2012; Dózsa-Farkas et al. 2015) were also found in the present study. It seems that these species are characteristic members of the Korean enchytraeid fauna, and that the genus *Hemienchytraeus* has a wide geographic distribution within the country. Fridericiacf.paroniana was found in this study and also in Mt. Hallasan, and the differences from *F.paroniana* were discussed in Dózsa-Farkas et al. (2018). At site 6, a species very similar to *F.sphaerica* Dózsa-Farkas et al., 2015 occurred, but according to the results of molecular analysis, it is different from *F.sphaerica.* Unfortunately, we found only two specimens from this putatively new species, and we will try to solve its taxonomic status later (therefore we referred to it now as F.cf.sphaerica).

**Table 2.** List of specimens used for molecular taxonomic analyses with collection data and GenBank accession numbers. Sequences determined in this study appear in bold. Paratype and holotype of the new species are indicated with P and H in parentheses, respectively.

Species	Collection information	Specimen ID	Genbank accession numbers		
			ITS	CO1	H3
*Achaetamultisacculata* sp. n.	Korea, site 9, 26.09.2016, coll. Y. Hong	1138	**MH128727**	**MH124584**	–
	Korea, site 8, 26.09.2016, coll. Y. Hong	1143	**MH128728**	**MH124585**	–
* Achaeta aberrans *	(see reference Erséus et al. 2010)	CE875	–	GU902030	–
* Achaeta affinis *	(see reference Dózsa-Farkas and Felföldi 2017)	919	KY583122	KY583145	–
* Achaeta bibulba *	(see reference Erséus et al. 2010)	CE1206	–	GU902031	–
* Achaeta bifollicula *		CE1035	–	GU902032	–
* Achaeta bohemica *	(see reference Dózsa-Farkas and Felföldi 2017)	849	KY583110	KY583128	–
* Achaeta camerani *		902	KY583126	KY583143	–
Achaeta cf. brevivasa	(see reference Erséus et al. 2010)	CE1234	–	GU902034	–
Achaeta cf. danica	(see reference Dózsa-Farkas and Felföldi 2017)	866	KY583118	KY583137	–
* Achaeta iberica *	(see reference Erséus et al. 2010)	CE1051	–	GU902036	–
* Achaeta koreana *	(see reference Dózsa-Farkas et al. 2018)	998	MG252199	–	–
* Achaeta macroampullacea *		1091	MG252200	MG252131	–
* Achaeta tothi *	(see reference Dózsa-Farkas and Felföldi 2017)	853	KY583113	KY583131	–
* Achaeta unibulba *		848	KY583109	KY583127	–
*Hemienchytraeus* sp. (outgroup)	(see references Erséus et al. 2010 and Dózsa-Farkas and Felföldi 2017)	CE1578	–	GU902080	–
		686	KY583108	–	
*Fridericiafloriformis* sp. n.	Korea, Gwaneumsa Trail, Mt. Hallasan, coordinates: 33.41667°N, 126.55000°E, 634 m asl., 27.10.2016, coll. Y. Hong.	1088	**MH128733**	**MH124586**	**MH124597**
	Korea, site 9, 26.09.2016, coll. Y. Hong	1133 (H)	**MH128730**	**MH124587**	–
		1134 (P)	**MH128731**	**MH124588**	–
		1136	**MH128729**	**MH124589**	**MH124598**
Fridericia cf. paroniana	Korea, Seongpanak Trail, Mt. Hallasan, coordinates: 33.37111°N, 126.56433°E, 1352 m asl., 17.08.2016, coll. Y. Hong	1056	–	**MH124590**	**MH124599**
		1057	–	**MH124591**	**MH124600**
		1148	–	**MH124592**	**MH124601**
		1152	–	**MH124593**	**MH124602**
Fridericia cf. sphaerica	Korea, site 6, 29.09.2016, coll. Y. Hong	1068	**MH128732**	**MH124594**	**MH124603**
* Fridericia cusanicaformis *	(see reference Dózsa-Farkas et al. 2015)	683	KR872373	KR872339	**MH124604**
* Fridericia dura *	(see references Dózsa-Farkas and Felföldi 2018 and Nagy et al. 2018)	907	MF547696	–	KX985894
* Fridericia galba *	(see reference Nagy et al. 2018)	1103	MF547697	MF547667	MF547688
		1123	MF547698	MF547668	MF547693
* Fridericia granulocyta *	(see reference Dózsa-Farkas et al. 2015)	672	KR872378	KR872344	KR872354
	(see reference Dózsa-Farkas et al. 2015 Korea, site 7, 29.09.2016	822	**MH128734**	**MH124595**	–
		1174	**MH128735**	**MH124596**	**MH124605**
* Fridericia peregrinabunda *	(see reference Dózsa-Farkas et al. 2015)	656	KR872375	KR872338	KR872351
* Fridericia ratzeli *	(see reference Dózsa-Farkas and Felföldi 2018)	844	KX985875	KX985884	KX985895
	(see reference Erséus et al. 2010)	CE782	–	GU902070	–
* Fridericia raxiensis *	(see reference Dózsa-Farkas and Felföldi 2018)	879	KX985868	MG921590	KX985885
* Fridericia regularis *	(see reference Nagy et al. 2018)	782	MF547703	–	MF547682
* Fridericia seoraksani *	(see reference Dózsa-Farkas et al. 2015)	679	KR872374	KR872340	KR872356
		823	KR872372	KR872342	KR872353
* Fridericia sphaerica *	(see reference Dózsa-Farkas et al. 2015)	820	KR872370	KR872334	KR872349
* Fridericia ventrochaetosa *	(see reference Nagy et al. 2018)	1114	MF547700	MF547676	MF547690
*Hemifridericiaparva* (outgroup)	(see reference Dózsa-Farkas and Felföldi 2015)	511a	KM591939	KM591923	KM591931

As mentioned above, the specimens of *Achaetamultisacculata* sp. n. did not possess any mature eggs, although the extraction of worms from soil samples was carried out several times from autumn to January. In contrast, *Achaetamacroampullacea* specimens mostly had mature eggs, so it can be assumed that *A.multisacculata* sp. n. belongs to that enchytraeid group where the worms reproduce only in certain seasons, as e.g. most *Mesenchytraeus* species (Dózsa-Farkas 1996).

Before 2007, the Korean enchytraeids were completely unknown. Results of subsequent studies (Dózsa-Farkas and Hong 2010; Christensen and Dózsa-Farkas 2012; Dózsa-Farkas et al. 2015; Dózsa-Farkas et al. 2018) indicated that the fauna is species rich. Including the findings in this paper, we described 23 species new for science and 13 new records for the Korean fauna. Thus, the Korean fauna now consists of 36 recorded terrestrial enchytraeid species. The high species number could be explained by the diverse geographic relief of the area which would result in many different microhabitats with differing microclimates, providing both for subtropical and temperate species (e.g., the typical tropical and subtropical *Hemienchytraeus* species or the widely distributed European *Fridericiabulboides* and *Hemifridericiaparva*) suitable conditions to flourish. We think that some worms are introduced species, e.g., the two terrestrial polychaetes, *Parergodrilusheideri* Reisinger, 1925 detected in a previous survey (Dózsa-Farkas and Hong 2010) and *Hrabeiellaperiglandulata* (a typical European taxon) which was detected in this study for the second time in Korea (only at site 8). Probably *Xetadrilusaphanus*, an enchytraeid species described from Brazil (Schmelz et al. 2011), is also an introduced species. Unfortunately, detailed biogeographical conclusions cannot be drawn yet regarding the Korean enchytraeid fauna, since the fauna of several areas has not been studied yet or is under study; furthermore some morphologically identical material (e.g., *Fridericiaseoraksani*, *F.sphaerica*), possibly representing cryptic species, requires further analysis.

## Supplementary Material

XML Treatment for Achaeta multisacculata

XML Treatment for Fridericia floriformis
